# Functional conservation of specialized ribosomes bearing genome-encoded variant rRNAs in *Vibrio* species

**DOI:** 10.1371/journal.pone.0289072

**Published:** 2023-12-05

**Authors:** Younkyung Choi, Eunkyoung Shin, Minho Lee, Ji-Hyun Yeom, Kangseok Lee

**Affiliations:** 1 Department of Life Science, Chung-Ang University, Seoul, Republic of Korea; 2 Department of Microbiology, College of Medicine, Hallym University, Chuncheon, Republic of Korea; Federal University Dutse, NIGERIA

## Abstract

Heterogeneity of ribosomal RNA (rRNA) sequences has recently emerged as a mechanism that can lead to subpopulations of specialized ribosomes. Our previous study showed that ribosomes containing highly divergent rRNAs expressed from the *rrnI* operon (I-ribosomes) can preferentially translate a subset of mRNAs such as *hspA* and *tpiA* in the *Vibrio vulnificus* CMCP6 strain. Here, we explored the functional conservation of I-ribosomes across *Vibrio* species. Exogenous expression of the *rrnI* operon in another *V*. *vulnificus* strain, MO6-24/O, and in another *Vibrio* species, *V*. *fischeri* (strain MJ11), decreased heat shock susceptibility by upregulating HspA expression. In addition, we provide direct evidence for the preferential synthesis of HspA by I-ribosomes in the *V*. *vulnificus* MO6-24/O strain. Furthermore, exogenous expression of *rrnI* in *V*. *vulnificus* MO6-24/O cells led to higher mortality of infected mice when compared to the wild-type (WT) strain and a strain expressing exogenous *rrnG*, a redundant rRNA gene in the *V*. *vulnificus* CMCP6 strain. Our findings suggest that specialized ribosomes bearing heterogeneous rRNAs play a conserved role in translational regulation among *Vibrio* species. This study shows the functional importance of rRNA heterogeneity in gene expression control by preferential translation of specific mRNAs, providing another layer of specialized ribosome system.

## Introduction

Recent growing evidence suggests that ribosomes are much more complex and heterogeneous macromolecules than previously thought [1–5]. Ribosome heterogeneity, caused by compositional changes such as divergent rRNAs, ribosomal proteins, ribosome-associated factors, or modifications of these factors, is found within species across various organisms. These subpopulations of ribosome variants are proposed to have specialized functions in regulating many biological processes—such as stress response, differentiation, and development—through the preferential translation of a specific subset of mRNAs [6–10]. Ribosomal heterogeneity due to variations in rRNA sequences has been observed in various organisms over several decades [11–14]. These genome-encoded heterogeneous rRNAs have been implicated in cellular development and adaptation to environmental changes. For instance, *Streptomyces coelicolor* differentially expresses heterogeneous rRNAs during the morphological developmental stages [15, 16], and heterogeneous 16S rRNAs in *Haloarcula marismortui* can be preferentially expressed under different growth temperatures [17]. In *Escherichia coli*, among seven rRNA operons, *rrnH* is highly expressed in response to nutrient limitation [6]. However, a critical question is whether the heterogeneity of rRNA sequences can lead to functional ribosome specialization.

Our previous experiments have shown that ribosome variants containing genome-encoded divergent rRNAs (I-ribosomes) can preferentially translate specific mRNAs, including *hspA* and *tpiA* mRNAs, in the opportunistic human pathogen *Vibrio vulnificus* CMCP6 [9]. Here, we investigated whether the physiological functions of I-ribosomes seen in *V*. *vulnificus* CMCP6 are conserved in other *Vibrio* strains and species.

## Materials and methods

### Ethics statement

All animal experiments were performed in accordance with the national guidelines for the use of animals in scientific research. The protocol was approved by Chung-Ang University Support Center (Approval No. CAU2012-0044). Per protocol, mice were sacrificed by using 100% CO_2_ followed by cervical dislocation if mice developed tumors greater than 1.8 cm in diameter, or if the mice showed any signs of persistent morbidity such as loss in weight greater than 20%, lethargy, unwillingness to ambulate, hunched posturing and ruffled fur. No invasive procedures likely to produce moderate to severe pain were performed. All efforts were made to minimize possible pain and suffering for mice during irradiation, monitoring, and euthanasia.

### Animals

7-week-old female ICR mice were kept under a 12:12 hours light/dark cycle, constant temperature of 25°C, 50% humidity, and allowed free access to water and a standard diet. Mouse body weight was monitored twice a week. Mice were acclimatized to the new environment for a few weeks before any experimental procedure.

### Bacterial culture conditions

All bacterial strains and plasmids used in this study are listed in S1 Table. *Vibrio* strains were grown at 30–45°C in LBS (Luria-Bertani [LB] medium containing NaCl at a final concentration of 2.5% [w/v]) under aerobic conditions. *E*. *coli* strains were grown in LB medium at 37°C. To express *rrnI* or *rrnG* exogenously, the pRK415-*rrnI* or pRK415-*rrnG* plasmid was conjugated into the *Vibrio* strains [18, 19].

### Isolation of crude ribosomes

Total RNA and crude ribosomes were isolated as previously described [9]. Briefly, cells grown to the mid-log phase were lysed using a French press, and the lysate was then subjected to loading onto a 30% sucrose cushion. Crude ribosomes were pelleted after centrifugation at 100,000g for 16 h at 4°C. The pellet was resuspended and loaded onto a 15–40% sucrose gradient and centrifuged at 79,500g for 13.5 h at 4°C. After centrifugation, 70S ribosomes were obtained by fractionation and measurement of the optical density (OD) at 260 nm. The quality and quantity of the extracted crude ribosomes were assessed using a Nanodrop 2000 spectrophotometer (Thermo Fisher Scientific, MA, USA).

### Quantification of I-rRNA in ribosomes

**Fig 1:**
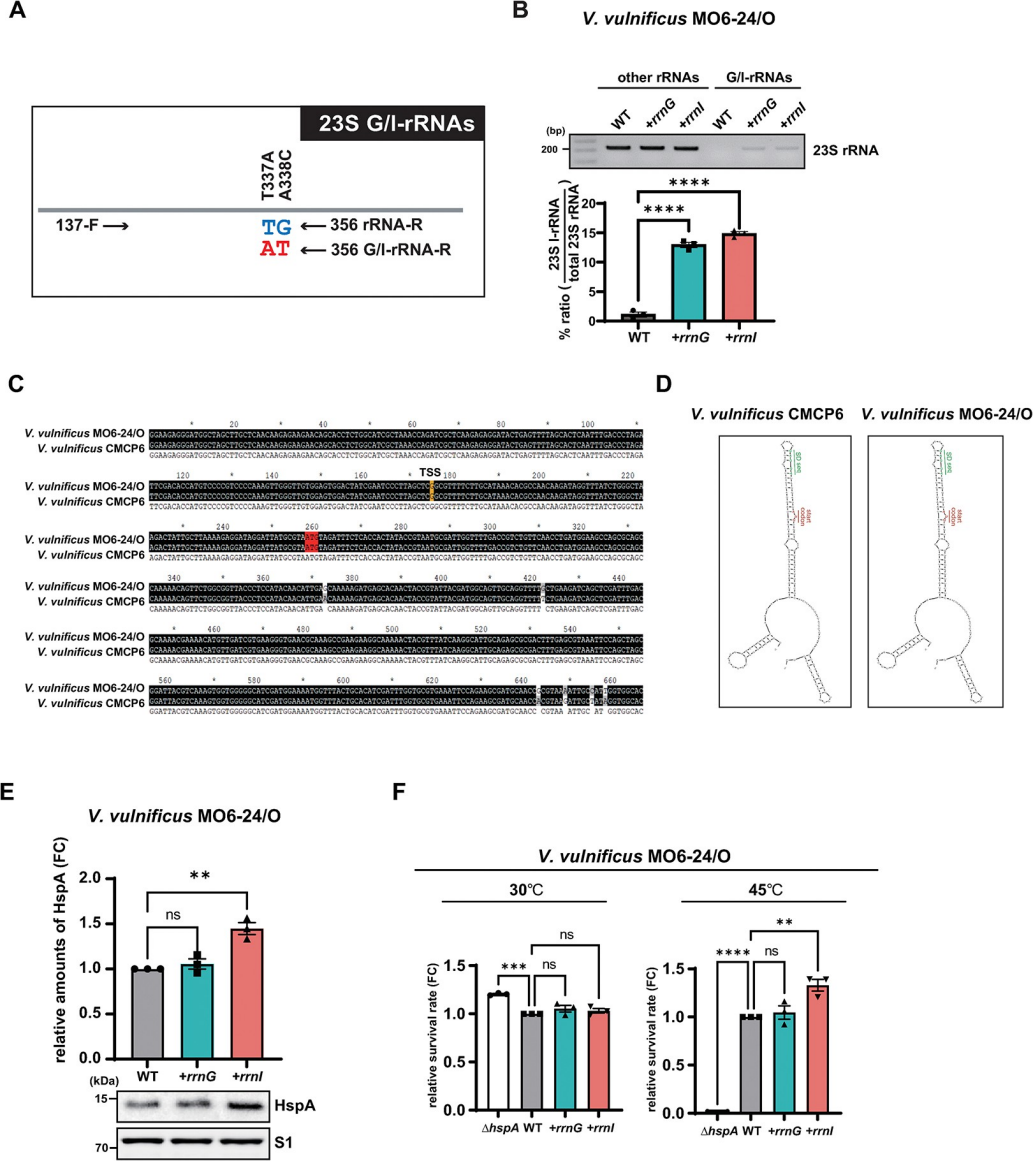
*rrnI*-dependent expression of HspA in *V*. *vulnificus* MO6-24/O strains. **(A)** Schematic representation of the allele-specific RT-PCR analysis analyzing the relative amounts of I-rRNA or G-rRNA. **(B)** The number of I-rRNA or G-rRNA amplicons and other rRNAs amplified from the cDNA of the MO6 WT, MO6+*rrnG*, and MO6+*rrnI* strains was determined by PCR using common and allele-specific primers. The cDNA was synthesized from rRNAs purified from crude ribosomes of these strains. PCR products were resolved on a 2% agarose gel. Data are presented as the mean ± SEM of three independent experiments. Statistical significance was determined using one-way ANOVA followed by Dunnett’s multiple comparison test (ns, not significant; ****, *P* < 0.0001). **(C)** Sequence alignment and secondary structure prediction of *hspA* mRNAs in the *V*. *vulnificus* strains MO6-24/O and CMCP6. Sequences of the *hspA* genes from *V*. *vulnificus* strains MO6-24/O and CMCP6 were obtained from the NCBI database (NC_014965.1 and NC_004459.2, respectively) and aligned using ClustalW (https://www.genome.jp/tools-bin/clustalw). The putative transcriptional start site (TSS) and the first nucleotide of the *hspA* gene are indicated in yellow and red, respectively. **(D)** Predicted secondary structures of *hspA* mRNAs from *V*. *vulnificus* CMCP6 (left) or *V*. *vulnificus* MO6-24/O (right). Secondary structures were obtained using the M-fold program (http://unafold.rna.Albany.edu). **(E)** *rrnI*-dependent expression of HspA. *Vibrio vulnificus* MO6-24/O strains (WT, *+rrnG*, and *+rrnI*) were grown in LBS at 30°C until mid-log phase and harvested for western blot analysis of HspA proteins using polyclonal antibodies against HspA. **(F)** Effect of *rrnI* expression on heat shock susceptibility of *V*. *vulnificus* MO6-24/O strains (WT, *+rrnG*, and *+rrnI*, and Δ*hspA*). The number of CFUs of each *V*. *vulnificus* MO6-24/O strain grown at 30°C or transiently grown at 45°C for 180 min was measured. Data are presented as the mean ± SEM of three independent experiments. Statistical significance was determined using one-way ANOVA followed by Dunnett’s multiple comparison test (ns, not significant; **, *P* < 0.01; ***, *P* < 0.001; ****, *P* < 0.0001).

The proportion of ribosomes incorporating I-rRNAs was determined by allele-specific RT-PCR as previously described [9, 20]. Complementary DNA (cDNA) was synthesized using an iScript cDNA synthesis kit (Bio-Rad Laboratories, CA, USA) according to the manufacturer’s instructions. Primers specific to the 23S *rrnI* or *rrnG* region were designed with a mismatch at a variable nucleotide position (Fig 1A). The PCR primers used for allele-specific RT-PCR are listed in S2 Table. All primers were purchased from BIONICS (Seoul, Korea).

### Western blot analysis

Cell lysates were prepared and subjected to 12% SDS polyacrylamide gel electrophoresis (SDS-PAGE) for subsequent immunoblotting with the appropriate antibodies. Images of the western blots were obtained using an Amersham Imager 600 (GE Healthcare Life Sciences, Amersham, Buckinghamshire, UK) and quantified using Quantity One (Bio-Rad Laboratories). The ribosomal protein S1 was used as the control. The following antibodies were used: anti-HspA (generated in our laboratory; [9]), anti-RNAP-β (Abcam, Cambridge, UK), and anti-S1 (obtained from the laboratory of Dr. Stanley N. Cohen [9]).

### Measurement of heat shock susceptibility

Heat shock susceptibility was measured as previously described [9]. Briefly, *Vibrio* strains were grown in LBS medium containing 0.2 μg/ml tetracycline at 30°C until reaching an OD_600_ of 0.6, and then each culture was immediately incubated in a water bath at 45°C for 180 min to induce heat shock.

### Co-immunoprecipitation

Co-immunoprecipitation of nascent peptides of HspA or RNAP-β subunit bound ribosomes was performed as previously described [20]. The presence of peptides in the eluted samples was confirmed by western blot, and total RNA was purified by ethanol precipitation. Purified RNA in the samples was quantified by allele-specific RT-PCR.

### Mouse mortality test

The virulence of *V*. *vulnificus* was measured using a mouse mortality test as previously described [9]. Briefly, the *V*. *vulnificus* MO6-24/O strains were grown in LBS medium to mid-log phase. Before injection, pathogen-free, 7-week-old female ICR mice (*n* = 10 mice per treatment) were fasted for 24 h and water deprived for 4 h. Tetracycline (200 mg per kg of body weight) was administered to the mice orally, and iron dextran (80 μg per g body weight) was injected intraperitoneally. After 1 h, *V*. *vulnificus* MO6-24/O (1 × 10^6^) cells were injected subcutaneously into the mice. Mice were monitored for 25 h, and dead mice were counted.

### Measurement of viable bacterial counts in mouse organs

Viable bacterial counts of *V*. *vulnificus* in mouse organs were performed as described previously [9]. Briefly, mice (*n* = 5 mice per treatment) were infected subcutaneously with *V*. *vulnificus* MO6-24/O. Mice were sacrificed 6 h post-infection, and spleens and livers were isolated. The organs were weighed, homogenized, and dissolved in LBS, and the homogenates were plated on LBS agar or LBS agar containing 0.2 μg/ml tetracycline and incubated overnight at 30°C. Bacterial colonies were then counted and expressed as the number of colony-forming units (CFUs)/g.

### Phylogenetic analysis of I-rRNA based on rRNA gene sequences

For the phylogenetic analysis of *Vibrio* species, 16S or 23S rRNA sequences were aligned using Silva (https://www.arb-silva.de/). Evolutionary distances were calculated using the Kimura two-parameter model [21]. The phylogenetic trees were constructed using the neighbor-joining method [22] in the MEGA7 program [23] with bootstrap values based on 1,000 replicates [24–26].

### Statistical analysis

All statistical details of experiments are included in the figure legends. Data are presented as the mean ± standard error of the mean (SEM). Statistical analyses were performed using Graph Pad Prism 9 software (version 9.4.1; Graph Pad Software Inc.), and *P*-values were obtained from a two-tailed unpaired *t*-test or one-way analysis of variance (ANOVA) followed by Dunnett’s multiple comparison test.

## Results

### Effect of *rrnI* expression on HspA levels in *V*. *vulnificus* MO6-24/O

To determine the effect of *rrnI* expression on HspA levels in a *V*. *vulnificus* strain that does not express highly variant rRNA genes, *V*. *vulnificus* MO6-24/O strains exogenously expressing *rrnI* (MO6+*rrnI*) or *rrnG* (MO6+*rrnG*) were constructed. The *rrnG* operon was used as a negative control to evaluate the effect of rRNA gene copy number variation. It is one of the redundant rRNA genes in the *V*. *vulnificus* CMCP6 strain [9]. The incorporation of exogenously expressed rRNA from the *rrnI* or *rrnG* operon (I-rRNAs or G-rRNAs) into ribosomes was assessed by allele-specific RT-PCR using primers specific to bind to 23S I-rRNA and G-rRNA (Fig 1A). These exogenously expressed rRNAs represented ~15% of the total rRNAs in these strains (Fig 1B). Similar results were obtained using the 23S I-rRNA-specific primers in allele-specific RT-PCR (S1 Fig).

Next, we investigated whether the HspA protein level was increased in MO6*+rrnI* cells. The sequences of the *hspA* gene were 99% identical between the *V*. *vulnificus* MO6-24/O and CMCP6 strains (Fig 1C), generating virtually the same secondary structure of *hspA* mRNA (Fig 1D). Expression of HspA was increased by approximately 1.5-fold in MO6*+rrnI* cells compared with the levels seen in MO6 WT and MO6+*rrnG* cells (Fig 1E). Consistent with previous results showing a strong correlation between heat shock susceptibility and the *rrnI* expression-dependent changes in HspA expression in the *V*. *vulnificus* CMCP6 strain [9], heat shock at 45°C for 60 min resulted in a ~30% increase in CFUs/g of the MO6+*rrnI* strain compared to those of the MO6 WT and MO6+*rrnG* strains (Fig 1F). These results indicate that the increased heat shock susceptibility of MO6+*rrnI* cells is a direct result of the overexpression of HspA in these cells.

### Preferential association of the I-ribosome to nascent HspA peptides in *V*. *vulnificus* MO6-24/O

**Fig 2:**
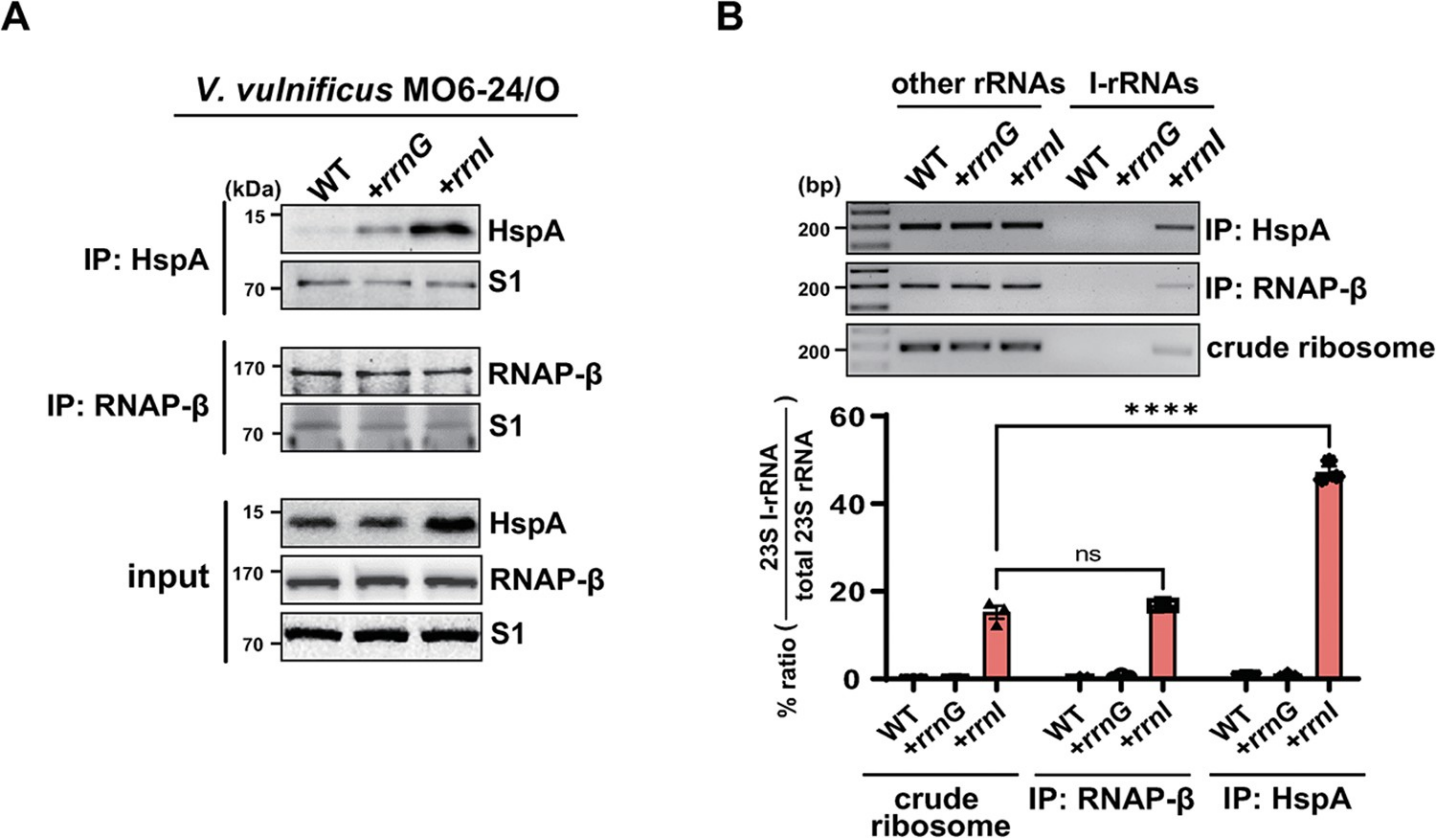
Co-immunoprecipitation (co-IP) showing the predominant association of I-ribosomes with HspA nascent peptides in *V*. ***vulnificus* MO6-24/O strains. (A)** Identification of HspA and the RNAP-β subunit after co-IP. The precipitate was subjected to western blotting with antibodies against HspA or RNAP-β. S1 protein was used as a loading control. **(B)** Characterization of the expression and assembly of I-rRNAs after co-IP. The number of amplicons of 23S I-rRNAs and other 23S rRNAs amplified from the cDNAs of the MO6 WT, MO6*+rrnG*, and MO6*+rrnI* strains was determined by RT-PCR using common and allele-specific primers. The cDNA was synthesized from rRNAs purified from immunoprecipitated samples or crude ribosome samples, which was used as controls. PCR products were resolved on a 2% agarose gel. Data are presented as the mean ± SEM of three independent experiments. Statistical significance was determined using one-way ANOVA followed by Dunnett’s multiple comparison test (ns, not significant; ****, *P* < 0.0001).

To explore whether *hspA* mRNA is preferentially translated by I-ribosomes in *V*. *vulnificus* MO6-24/O cells, co-immunoprecipitation (co-IP) analysis using anti-HspA antibodies was performed in MO6 WT, MO6+*rrnI*, and MO6+*rrnG* cells [20]; Fig 2A). Immunoprecipitation of RNAP-β, levels of which are not *rrnI* expression dependent, was used as a control.

Total rRNA was purified from the co-immunoprecipitated ribosomes and relative amounts of I-rRNA in the total rRNA populations were measured by allele-specific RT-PCR. In MO6+*rrnI* cells, I-rRNA accounted for ~50% of the total rRNA from HspA-bound ribosomes, whereas it represented ~15% of the total rRNAs from crude ribosome samples or RNAP-β-bound ribosomes (Fig 2B). This suggests that *hspA* mRNA can be preferentially translated by I-ribosomes in the *V*. *vulnificus* MO6-24/O strain.

### Effect of *rrnI* on *V*. *vulnificus* MO6-24/O virulence in mice

**Fig 3:**
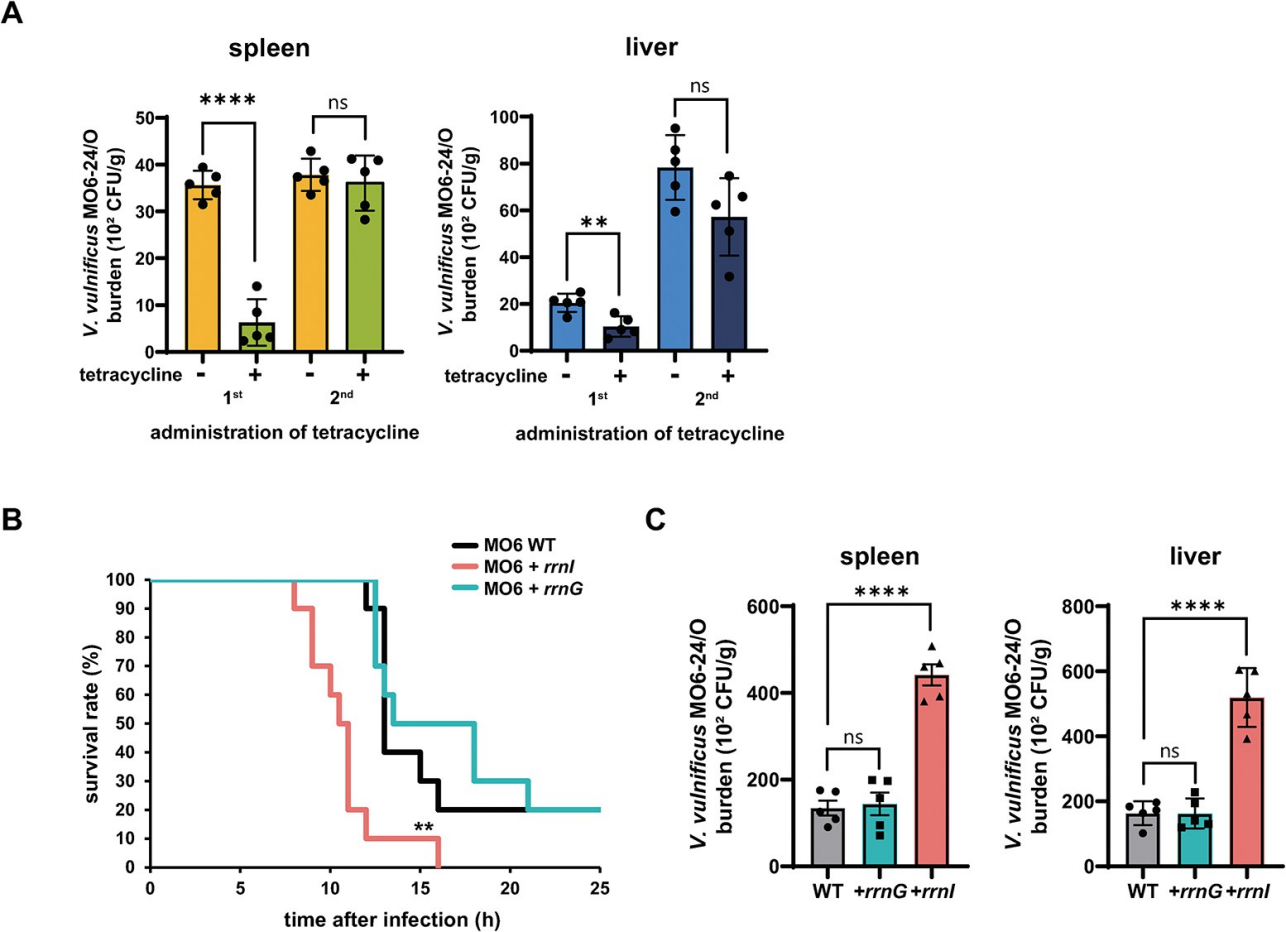
Effect of *rrnI* expression on the virulence of *V*. ***vulnificus* MO6-24/O in mice. (A)** Plasmid loss rate of *V*. *vulnificus* MO6-24/O in mice. Pathogen-free 7-week-old female ICR mice that were pretreated with tetracycline and iron dextran (*n* = 5 mice per group) were subcutaneously injected with 1 × 10^6^ cells of *V*. *vulnificus* MO6-24/O (MO6 WT). Mice with tetracycline administered orally before infection and those treated once more 6 h after the initial treatment were sacrificed 6 h and 12 h after infection, respectively. Data are presented as the mean ± SEM of three independent experiments. Statistical significance was determined using a two-tailed unpaired Student’s t-test (ns, not significant; ****, *P* < 0.0001). **(B)** Survival rates and the survival time of mice infected with *V*. *vulnificus* MO6-24/O strains (MO6 WT, MO6*+rrnG*, and MO6*+rrnI*). Pathogen-free 7-week-old female ICR mice pretreated with iron dextran (*n* =  10 mice per group) were intraperitoneally injected with 8 × 10^2^ cells of the *V*. *vulnificus* MO6-24/O strains. Survival rate was monitored for 25 h. Data are presented as the mean ± SEM of three independent experiments. Two-tailed unpaired Student’s *t*-tests were used to assess significant differences: ** denotes *P* < 0.01 for comparisons of MO6*+rrnI*-infected mice versus WT- or MO6*+rrnG-*infected mice. **(C)** Viable bacterial counts in the spleen and liver of mice infected with the *V*. *vulnificus* MO6-24/O strains (MO6 WT, MO6+*rrnG*, and MO6+*rrnI*). Six hours after bacterial infection, the mice (*n* = 5 mice per group) were sacrificed. Results are expressed as numbers of CFU/g of each organ. Data are presented as the mean ± SEM of three independent experiments. Statistical significance was determined using one-way ANOVA followed by Dunnett’s multiple comparison test (ns, not significant; ****, *P* < 0.0001).

In our previous study, the survival rates and duration of survival of mice infected with *rrnI*-deleted *V*. *vulnificus* CMCP6 cells were increased compared to that of mice infected with the CMCP6 WT strain [9]. To confirm the physiological function of I-ribosomes in the virulence of *V*. *vulnificus* MO6-24/O, the MO6 WT, MO6+*rrnG*, or MO6+*rrnI* strains were intraperitoneally injected into BALB/c mice, and the survival rate and duration of infected mice were examined. In addition, since plasmid vectors require antibiotics for maintenance in *V*. *vulnificus* cells after being infected into mice [27], we first confirmed the plasmid loss rate of WT *V*. *vulnificus* MO6-24/O by comparing the number of CFUs isolated from representative mice organs (spleen and liver) after administration of tetracycline. As shown in Fig 3A, the plasmid loss rate was ~82% and ~49% after the first administration of antibiotics in the spleen and liver, respectively (Fig 3A). After the second administration of antibiotics, the plasmid loss rate was reduced in both organs (Fig 3A), suggesting that two consecutive administrations of antibiotics are needed to maintain plasmid vectors in *V*. *vulnificus* cells in infected mice. Thus, we used two consecutive injections of tetracycline for vector maintenance in the mouse mortality experiment. Lower survival rates were observed in MO6*+rrnI*-infected mice than in mice infected with the MO6 WT or MO6*+rrnG* strains (Fig 3B), indicating that I-rRNA influences the virulence of *V*. *vulnificus* MO6-24/O.

Next, we measured the number of viable bacterial cells in the spleen and liver of mice infected with the MO6 WT, MO6+*rrnG*, or MO6+*rrnI* strains to further explore the effect of *rrnI* expression on bacterial survival in the host environment. We administered tetracycline twice and sacrificed the mice 6 h after bacterial infection, a time chosen based on the mouse survival duration. A ~3-fold increase in colonization was observed in the organs of the MO6*+rrnI* strain-infected mice compared to the MO6 WT strain-infected mice (Fig 3C). By contrast, no significant changes in colonization were detected between mice infected by the MO6*+rrnG* strain and the MO6 WT strain (Fig 3C), suggesting that MO6+*rrnI* cells can grow more rapidly than the other strains in mouse organs. These results indicate that expression of I-rRNA makes *V*. *vulnificus* more virulent in mice.

### Effect of *rrnI* expression on HspA levels in *V*. *fischeri* MJ11

**Fig 4:**
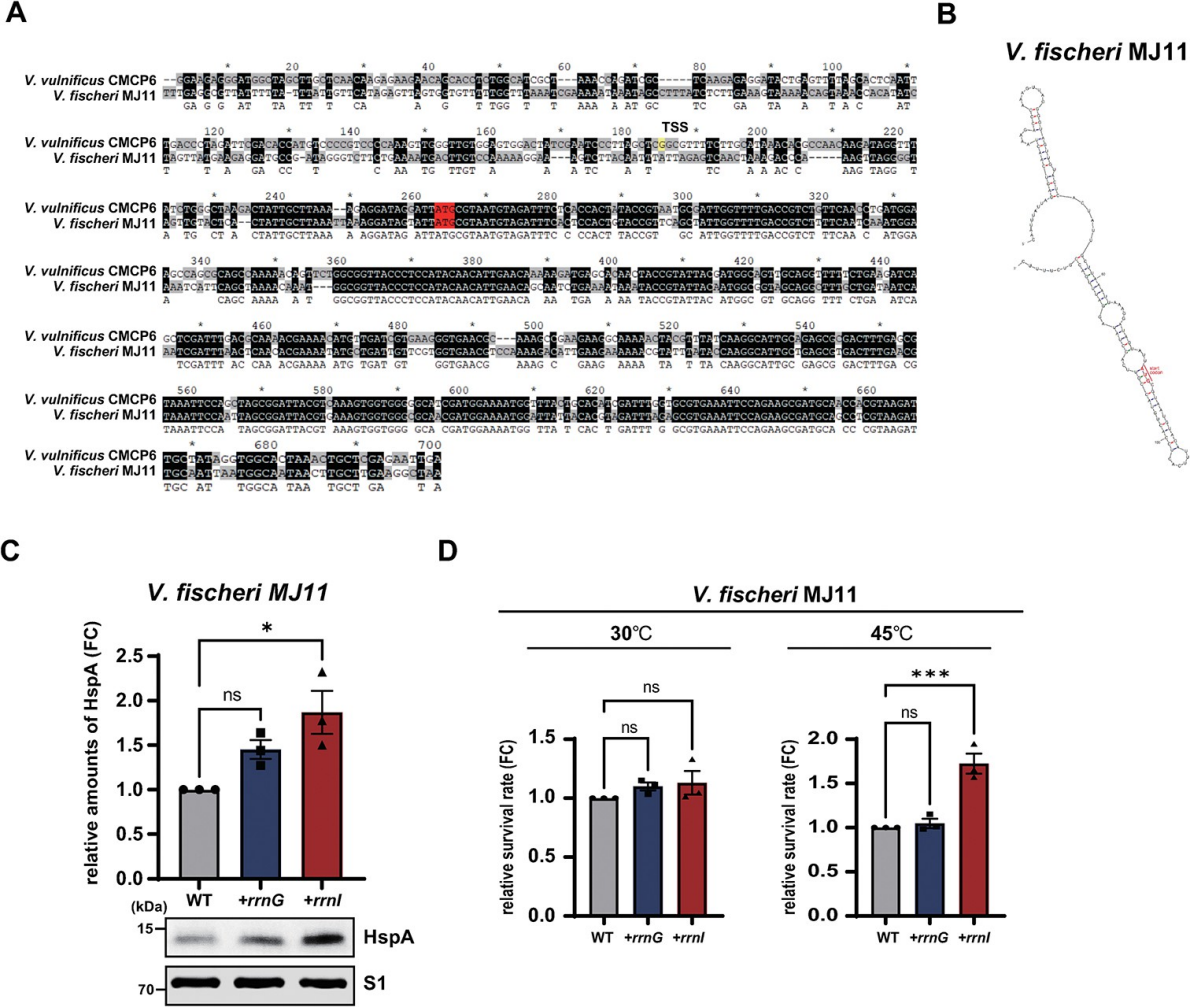
*rrnI*-dependent expression of HspA in *V*. *fischeri* MJ11 strains. **(A)** Sequences of *hspA* genes in *V*. *fischeri* MJ11 and *V*. *vulnificus* CMCP6 were obtained from the NCBI database (NC_011184.1 and NC_004459.2, respectively) and were aligned using ClustalW. The putative transcriptional start site of *V*. *vulnificus* CMCP6 and the first nucleotide of the *hspA* sequence are indicated in yellow and red, respectively. (**B**) Prediction of the secondary structures of *V*. *fischeri* MJ11 *hspA* mRNAs. Secondary structures were obtained using the M-fold program. **(C)** *V*. *fischeri* MJ11 strains (WT, *+rrnG*, and *+rrnI*) were grown and harvested for a western blot analysis of HspA proteins using polyclonal antibodies against HspA (see the Fig 1E legend for details). **(D)** Effect of *rrnI* expression on heat shock susceptibility of *V*. *fischeri* MJ11 strains (WT, *+rrnG*, and *+rrnI*). The number of CFUs of each *V*. *fischeri* MJ11 strain was measured as described in the legend of Fig 1F. The expression levels of HspA and the number of CFUs were compared by setting those of the WT to 1. Data are presented as the mean ± SEM of three independent experiments. Statistical significance was determined using one-way ANOVA followed by Dunnett’s multiple comparison test (ns, not significant; *, *P* < 0.05 and ***, *P* < 0.001).

To determine the effect of *rrnI* expression on HspA expression in another *Vibrio* species that does not express highly variant rRNA genes, *V*. *fischeri* MJ11 strains exogenously expressing *rrnI* (MJ11+*rrnI*) or *rrnG* (MJ11+*rrnG*) were constructed. In this *Vibrio* species, the *hspA* gene has 86% sequence homology with that of the *V*. *vulnificus* CMCP6 strain (Fig 4A), resulting in a secondary structure of *hspA* mRNA that is distinct from that of the *V*. *vulnificus* strains (Fig 4B).

We also observed enhanced expression of HspA and heat shock tolerance in the MJ11+*rrnI* strain compared to those in the MJ11 WT and MJ11+*rrnG* strains (Fig 4C and 4D). These results suggest that exogenous expression of *rrnI* enhances HspA expression, leading to the increased heat shock resistance of *V*. *fischeri* cells.

## Discussion

Emerging studies have suggested that heterogeneous rRNAs have specialized roles in protein synthesis. However, a longstanding question is how ribosome heterogeneity at the level of rRNAs can contribute to specialized ribosome function. Our previous studies showed that ribosomes containing the most divergent rRNAs preferentially bind and translate specific stress-response mRNAs in *V*. *vulnificus* CMCP6 cells under stress conditions [9, 20]. Our present study highlights the functional conservation of these divergent rRNAs in other *Vibrio* species that do not express highly variant rRNAs. Specifically, exogenous expression of I-rRNAs in the *V*. *vulnificus* MO6-24/O strain resulted in enhanced HspA expression, heat shock tolerance, and virulence (Figs 1, 3 and S1 Fig). In addition, the co-immunoprecipitation analysis shows that the enhanced HspA expression is a direct consequence of I-ribosome-mediated preferential translation of *hspA* mRNA (Fig 2). As previously proposed, the target mRNAs of the I-ribosome showed a common feature [9]. In the case of *hspA* mRNA in *V*. *fischeri*, the putative Shine-Dalgarno (SD) sequence is also embedded in the secondary structure (Fig 4B), indicating that I-ribosome mediated mRNA selection does not require a strong SD/anti-SD interaction.

**Fig 5:**
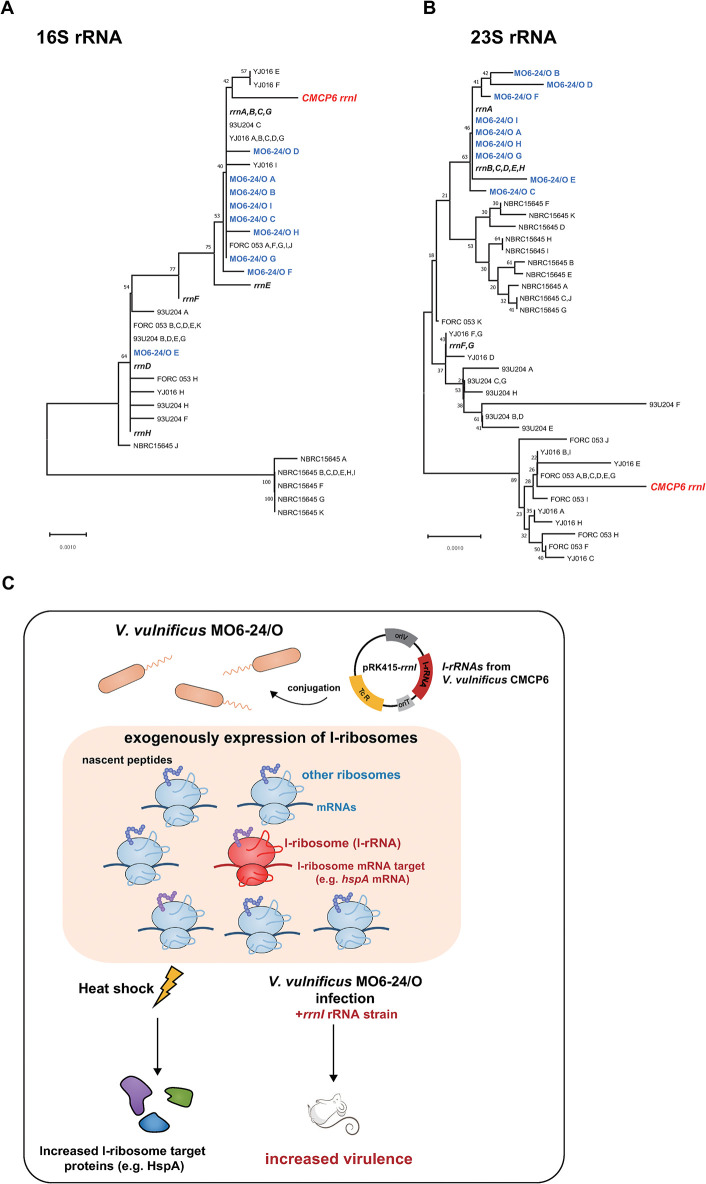
The role of the I-ribosome in bacterial survival. **(A** and **B)** Phylogenetic trees of rRNA operons in *V*. *vulnificus* strains. Neighbor-joining phylogenetic trees based on 16S rRNA **(A)** and 23S rRNA **(B)** sequences showing the positions of rRNA operons within the *Vibrio* genomes. Bold text represents the *V*. *vulnificus* CMCP6 rRNA operon. *V*. *vulnificus* strains that contain rRNA genes similar to the *rrnI* operon were selected using NCBI nucleotide blast (https://blast.ncbi.nlm.nih.gov) and the rRNA gene sequences of these strains were obtained from the NCBI database. The rRNA genes of each strain were named starting with “A” according to the nucleotide position in the genome. The values above and below the branches (expressed as percentages) indicate the robustness of the corresponding branch as determined by bootstrap analysis (heuristic search). **(C)** I-ribosomes from *V*. *vulnificus* CMCP6 were exogenously expressed in *V*. *vulnificus* MO6-24/O strains. Overexpression of *rrnI* in the *V*. *vulnificus* MO6-24/O strain leads to increased bacterial survival in heat shock and host environments by preferentially translating the target mRNAs.

A comparative analysis of the 16S and 23S rRNA gene sequences of *Vibrio* strains indicates that *rrnI* operon from *V*. *vulnificus* CMCP6 is not closely related to rRNA operons of the other *Vibrio* species (Fig 5A and 5B), which suggests that the *rrnI* operon might have evolved recently in the *V*. *vulnificus* CMCP6 strain.

Taken together, our findings indicate that heterogeneous ribosomes differing in their rRNA sequences have evolved to have a unique and conserved function in selectively translating specific mRNAs in *Vibrio* species (Fig 5C). Further studies are needed to unveil why some bacterial species have retained an evolutionarily unfavorable pathway of maintaining divergent rRNA genes to control protein synthesis from specific mRNAs.

## Conclusions

This study provides evidence for the functional conservation of ribosomes carrying heterogeneous rRNAs among *Vibrio* species. We observed that the exogenous expression of the *rrnI* operon from *V*. *vulnificus* CMCP6 led to enhanced expression of HspA by preferential binding of I-ribosomes to *hspA* mRNA, and, consequently, decreased heat shock susceptibility in *V*. *vulnificus* MO6-24/O strain and *V*. *fischeri*. This study highlights functional conservation of specialized ribosomes in preferential translation of specific mRNAs among *Vibrio* species and provides another layer of regulation of gene expression at the ribosome level.

## Supporting information

S1 Fig *rrnI*-dependent expression of HspA in *V*. *vulnificus* MO6-24/O strains. **(A)** Schematic representation of the allele-specific RT-PCR analysis analyzing the relative amounts of I-rRNA. **(B)** The number of I-rRNA amplicons and other rRNAs amplified from the cDNA of the MO6 WT, MO6+*rrnG*, and MO6+*rrnI* strains was determined by PCR using common and allele-specific primers. The cDNA was synthesized from rRNAs purified from crude ribosomes of these strains. PCR products were resolved on a 2% agarose gel. Data are presented as the mean ± SEM of three independent experiments. Statistical significance was determined using one-way ANOVA followed by Dunnett’s multiple comparison test (ns, not significant; ****, *P* < 0.0001).
(TIF)

S1 Table Bacterial strains and plasmids used in this study [9, 28, 29]. (DOCX)

S2 Table Primers used in this study. (DOCX)

S1 Dataset (XLSX)

S1 Raw images (PDF)
