# Supplementary figures and images for: Functional conservation of specialized ribosomes bearing genome-encoded variant rRNAs in Vibrio species
Source: PLoS One. 2023 Dec 5;18(12):e0289072. doi: 10.1371/journal.pone.0289072 (PMC10697612; doi:10.1371/journal.pone.0289072)

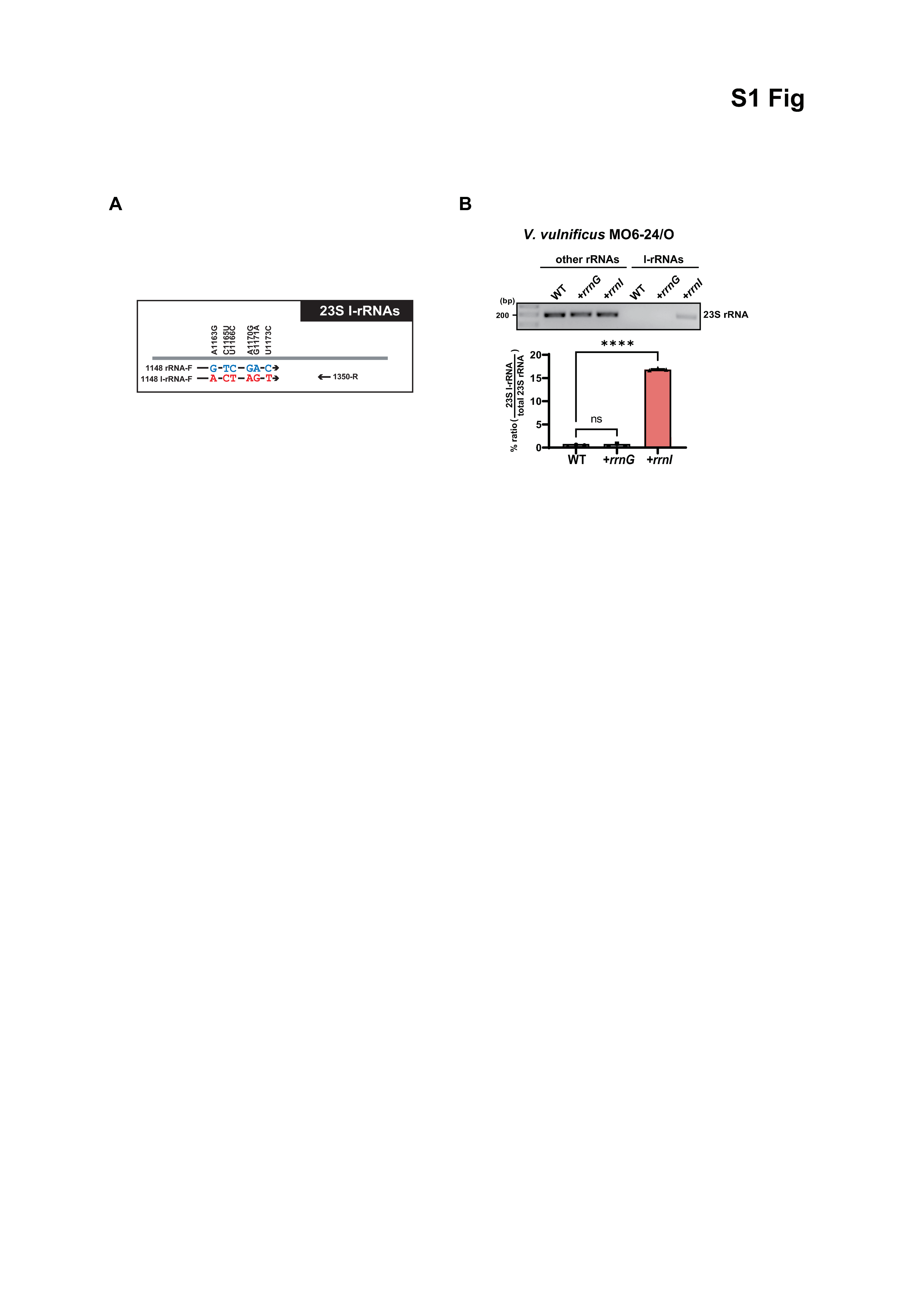

Supplement: S1 Fig — (A) Schematic representation of the allele-specific RT-PCR analysis analyzing the relative amounts of I-rRNA. (B) The number of I-rRNA amplicons and other rRNAs amplified from the cDNA of the MO6 WT, MO6+rrnG, and MO6+rrnI strains was determined by PCR using common and allele-specific primers. The cDNA was synthesized from rRNAs purified from crude ribosomes of these strains. PCR products were resolved on a 2% agarose gel. Data are presented as the mean ± SEM of three independent experiments. Statistical significance was determined using one-way ANOVA followed by Dunnett’s multiple comparison test (ns, not significant; ****, P < 0.0001). (TIF) [file pone.0289072.s001.tif]

Fig 1B

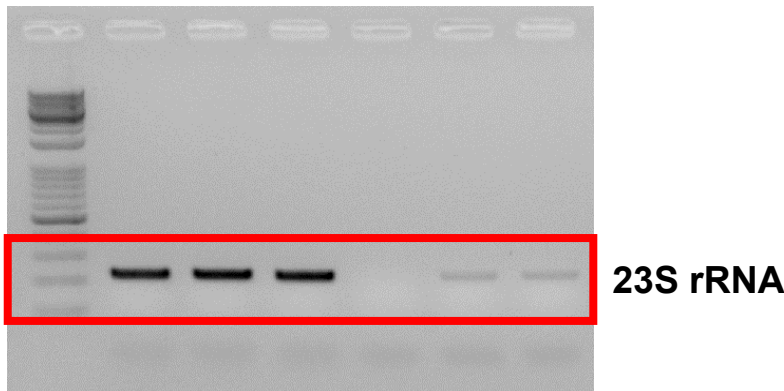

Fig 1C

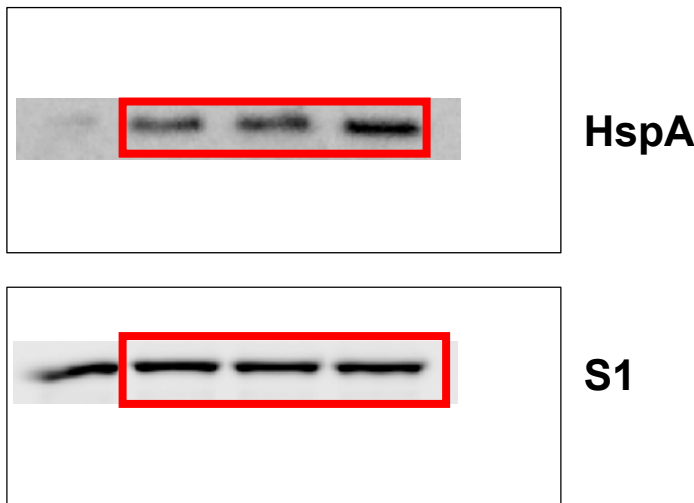

Fig 2A

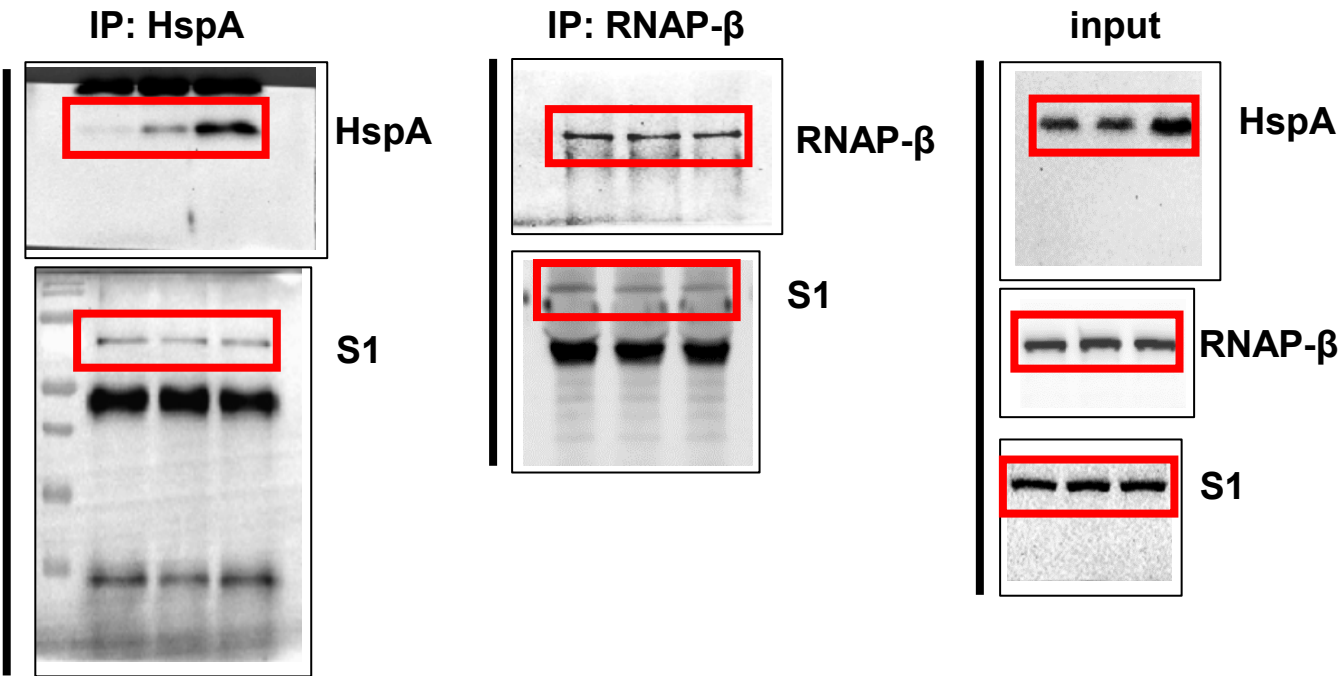

Fig 2B

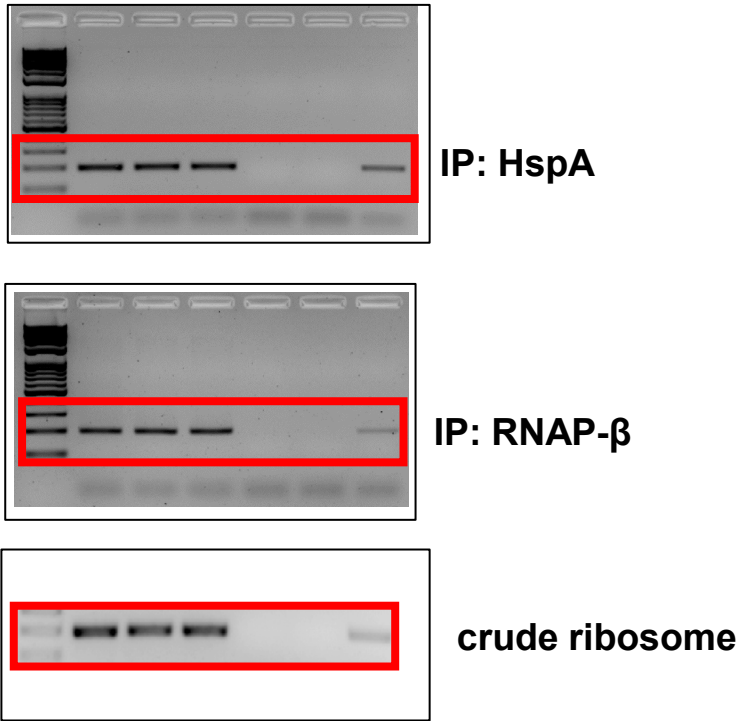

Fig 4C

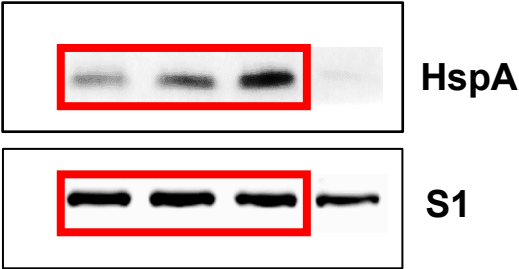

S1 Fig B

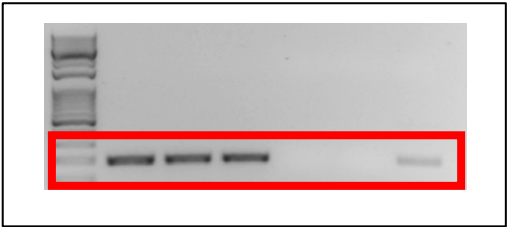

23S rRNA

Supplement: S1 Raw images — (PDF) [file pone.0289072.s005.pdf]
