# Supplementary material for: Functional conservation of specialized ribosomes bearing genome-encoded variant rRNAs in Vibrio species
Source: PLoS One. 2023 Dec 5;18(12):e0289072. doi: 10.1371/journal.pone.0289072 (PMC10697612; doi:10.1371/journal.pone.0289072)
Supplement: S1 Table — (DOCX) [file pone.0289072.s002.docx]

**S1 Table. Bacterial strains and plasmids used in this study**

| **Strains or Plasmids** | **Relevant characteristics** | **References** |
| --- | --- | --- |
| ***Vibrio* species** | | |
| *V. vulnificus* MO6-24/O | Clinical isolate | [28] |
| *V. fischeri* MJ11 | Clinical isolate | [29] |
| ***Escherichia coli*** | | |
| SM10λ*pir* | *thi-1 thr leu tonA lacY supE recA*::Rp4-2-Tc::Mu λ*pir,* Km^R^ | [9] |
| **Plasmids** | | |
| pRK415 | RK2-derived *oriV,* Tn^R^ | [9] |
| pRK415-rrnI | pRK415 containing the *rrnI* operon | [9] |
| pRK415-rrnG | pRK415 containing the *rrnG* operon | [9] |
