# Supplementary material for: Functional conservation of specialized ribosomes bearing genome-encoded variant rRNAs in Vibrio species
Source: PLoS One. 2023 Dec 5;18(12):e0289072. doi: 10.1371/journal.pone.0289072 (PMC10697612; doi:10.1371/journal.pone.0289072)
Supplement: S2 Table — (DOCX) [file pone.0289072.s003.docx]

S2 Table. Primers used in this study

| **Primers** | **Sequences** |
| --- | --- |
| 23S-1148 rRNA-F | 5′-TGCGGCAATGTTCTTTGAAC-3′ |
| 23S-1148 I-rRNA-F | 5′-TGCGGCAATATCTTTTAGAT-3′ |
| 23S-rRNA-1350R | 5′-CGGCCTCGCCTTAGGGGTCG-3′ |
| 23S-137 rRNA-F | 5′-ATAAGCCAGTATCATTGAGT-3′ |
| 23S-356 rRNA-R | 5′-TTTCACTGAACATGCATCGT-3′ |
| 23S-356 G/I-rRNA-R | 5′-TTTCACTGAACATGCATCTA-3′ |
